# Epidemiological Characterization of Group B Streptococcus Infections in Alberta, Canada: An Update from 2014 to 2020

**DOI:** 10.1128/Spectrum.01283-21

**Published:** 2021-11-17

**Authors:** Angela Ma, L. Alexa Thompson, Thomas Corsiatto, Donna Hurteau, Gregory J. Tyrrell

**Affiliations:** a Division of Diagnostic and Applied Microbiology, Department of Laboratory Medicine and Pathology, University of Alberta, Edmonton, Canada; b Alberta Precision Laboratories—Provincial Laboratory for Public Health, Edmonton, Canada; Children’s Hospital Los Angeles, University of Southern California

**Keywords:** antibiotic resistance, capsule type, early-onset disease, group B *Streptococcus*, late-onset disease

## Abstract

Group B Streptococcus (GBS) is a leading cause of invasive neonatal disease. Epidemiological surveillance of GBS is important to determine cumulative incidence, antimicrobial resistance rates, and maternal and neonatal disease prevention. In this study, we present an update on GBS epidemiology in Alberta, Canada, from 2014 to 2020. Over the 7-year period, 1,556 GBS isolates were submitted to the Alberta Public Health Laboratory for capsular polysaccharide (CPS) typing and antimicrobial susceptibility testing. We analyzed the distribution of CPS types in Alberta and found CPS types III (23.6%), Ia (16.0%), Ib (14.8%), II (13.3%), V (12.7%), IV (12.5%), and VI (2.38%) to be the most prevalent. Less than 1% each of CPS types VII, VIII, and IX were identified. In agreement with historical data, the presence of CPS type IV continued to rise across Alberta, particularly in cases of adult infection, where a 2-fold increase was observed. Cumulative incidences of GBS cases per 100,000 population and late-onset disease per 1,000 live births increased from 4.43 to 5.36 and 0.38 to 0.41, respectively, from 2014 to 2020. However, the incidence of early-onset disease decreased during the 7-year period from 0.2 to 0.07, suggestive of successful intrapartum chemoprophylaxis treatment programs. All GBS isolates were susceptible to penicillin and vancomycin. However, nonsusceptibility to erythromycin increased significantly, from 36.85% to 50.8%, from 2014 to 2020. Similarly, nonsusceptibility to clindamycin also increased significantly, from 21.0% to 45.8%. In comparison to historical data, the overall rates of GBS infection and antimicrobial resistance have increased and the predominant CPS types have changed.

**IMPORTANCE** This work describes the epidemiology of invasive infections caused by the bacterium group B Streptococcus (GBS) in Alberta, Canada. We show that rates of invasive GBS disease have increased from 2014 to 2020 for both adult disease and late-onset disease in neonates, whereas the rate of early onset disease in neonates has decreased. We also show that the rate of resistance to erythromycin (an antibiotic used to treat GBS) has also increased in this time.

## INTRODUCTION

The Gram-positive bacterium Streptococcus agalactiae, also known as group B Streptococcus (GBS), is a prominent causative agent of disease, particularly in newborns (neonates), the elderly, and immunocompromised adults (1–4). As facultative anaerobes, GBS organisms in healthy adults are mainly found as commensal organisms in the gastrointestinal and urogenital tracts (5–7). However, GBS bacteria are known to be one of the leading causes of infectious disease in neonates in North America (8). Neonatal acquisition of GBS is primarily a result of vertical transmission from colonized mothers to neonates during pregnancy or birth (9, 10). Through the activation of various virulence factors, GBS can cause sepsis, pneumonia, or meningitis in neonates, leading to severe disease (11). GBS-associated disease in infants can occur within the first 7 days of life (early-onset disease [EOD]) or within 8 to 90 days after birth (late-onset disease [LOD]) (12, 13). In adults, invasive GBS infections can also result in meningitis, endocarditis, pneumonia, and deep tissue infections (14, 15).

GBS bacteria possess an exterior capsular polysaccharide (CPS) that has traditionally been used to aid in classifying GBS through designation with 1 of the 10 different CPS serotypes (16), but there continue to be nontypeable GBS cases reported where the CPS type cannot be determined (17–19). Classification is important, as virulence and pathogenesis caused by GBS have been associated with specific CPS types (20, 21). Specifically, it has been shown that CPS type III GBS is more commonly implicated in neonatal disease (1) than GBS with CPS types Ia and V, which are major causative agents of invasive disease in adults (22, 23). The distribution of CPS types varies geographically, and their circulation within a specific region is also dynamic (1, 24). As a result of this variability, it is important to track and monitor the CPS type distribution and invasiveness to provide better insight regarding the prediction and prevention of human disease.

In a previous GBS epidemiological study conducted in Alberta, we described an overall increasing trend in the incidence of invasive GBS infection for all age groups and an increase in EOD and LOD incidence from 2003 to 2013 (25). In Canada, EOD prevention is accomplished by utilizing culture-based screening for pregnant mothers who are between 35 and 37 weeks of gestation (26). Canadian recommendations from the Society of Obstetricians and Gynaecologists for screening of GBS at 35 to 37 weeks were first published in 1997 (27). If the culture is positive for GBS or the mother previously had a newborn infected with GBS, it is recommended that intravenous antibiotic chemoprophylaxis be performed near the initiation of labor. This form of treatment aids in preventing GBS vertical transmission during birth and thus prevents colonization of the neonate with potentially virulent GBS. The chemoprophylactic antibiotic of choice is typically a penicillin compound, as GBS shows high susceptibility to this class of antimicrobial agents and penicillin susceptibility has remained relatively stable over time (3, 6). However, over the past 20 years, antimicrobial-resistant GBS isolates have increased, particularly isolates with resistance to macrolide antibiotics like erythromycin (28). This macrolide resistance is concerning, as mothers at risk for penicillin allergy anaphylaxis are typically administered erythromycin or clindamycin as second-line prophylactic treatments.

The surveillance of GBS, EOD, and LOD cumulative incidence and antimicrobial susceptibility provides vital information that can help guide the treatment and prevention of GBS-related disease. In this study, we aim to provide an update on the epidemiology of GBS in Alberta, Canada, from 2014 to 2020 by analyzing CPS serotype distribution across patient demographics, GBS incidence, and antimicrobial resistance rates.

## RESULTS

### Descriptive statistics of CPS type by patient demographics and comparison to historical data on GBS isolates.

Table 1 describes the distribution of CPS types and their descriptive statistics according to year and patient demographics, including age group and sex. Among 1,556 GBS isolates for which CPS typing was performed during the 7-year study period, CPS type III (23.6%) was the most prominent CPS type identified overall (*P* < 0.001). CPS types VII (0.19%), VIII (0.45%), and IX (0.13%) were the least frequently identified CPS types. Sixty-one GBS isolates (3.92%) were nontypeable for a CPS type. CPS type III GBS isolates were the most commonly found in each calendar year (*P* < 0.001) except 2019, when type Ib was the most frequently identified. Among GBS isolates obtained from cases of infection in EOD, LOD, children, and adults between 15 and 50 years of age, CPS type III was the most common (*P* < 0.001), accounting for 30.0%, 69.4%, 44.4%, and 19.1% of the respective age groups. CPS type Ia (17.3%) was the most commonly identified CPS type in adults over 50 years of age, with CPS types III (16.9%) and Ib (15.9%) following closely. Among males and females, CPS type III was again the most common CPS type identified (*P* < 0.001). One GBS isolate determined to be CPS type V was obtained from a patient of unknown sex.

**TABLE 1:** Descriptive statistics of CPS types in GBS isolates by patient demographics

Parameter (total no. of isolates)	No. (%) of isolates of CPS type:											*P* value
	Ia	Ib	II	III	IV	V	VI	VII	VIII	IX	NT^*a*^	
Overall (*n* = 1,556)	249 (16.0)	230 (14.8)	207 (13.3)	367 (23.6)	195 (12.5)	198 (12.7)	37 (2.38)	3 (0.19)	7 (0.45)	2 (0.13)	61 (3.92)	<0.001*^b^
Yr of isolation												<0.001*
2014 (*n* = 181)	25 (13.8)	34 (18.8)	20 (11.0)	47 (26.0)	17 (9.39)	18 (9.94)	3 (1.66)	1 (0.55)	0 (0)	ND^*c*^	16 (8.84)	
2015 (*n* = 227)	44 (19.4)	23 (10.1)	32 (14.1)	49 (21.6)	27 (11.9)	28 (12.3)	4 (1.76)	0 (0)	0 (0)	ND	20 (8.81)	
2016 (*n* = 206)	31 (15.1)	27 (13.1)	20 (9.71)	53 (25.7)	26 (12.6)	20 (9.71)	7 (3.40)	0 (0)	0 (0)	ND	22 (10.7)	
2017 (*n* = 211)	30 (14.2)	27 (12.8)	25 (11.9)	61 (28.9)	30 (14.2)	29 (13.7)	5 (2.37)	1 (0.47)	1 (0.47)	1 (0.47)	1 (0.47)	
2018 (*n* = 243)	34 (14.0)	33 (13.6)	37 (15.2)	53 (21.8)	40 (16.5)	35 (14.4)	8 (3.29)	0 (0)	2 (0.82)	0 (0)	1 (0.41)	
2019 (*n* = 251)	40 (15.9)	51 (20.3)	39 (15.5)	49 (19.5)	28 (11.2)	35 (13.9)	6 (2.39)	0 (0)	2 (0.80)	0 (0)	1 (0.40)	
2020 (*n* = 237)	45 (19.0)	35 (14.8)	34 (14.4)	55 (23.2)	27 (11.4)	33 (13.9)	4 (1.69)	1 (0.42)	2 (0.84)	1 (0.42)	0 (0)	
Age group												<0.001*
EOD (*n* = 50)	10 (20.0)	7 (14.0)	6 (12.0)	15 (30.0)	5 (10.0)	5 (10.0)	1 (2.00)	0 (0)	0 (0)	0 (0)	1 (2.00)	
LOD (*n* = 163)	21 (12.9)	13 (7.98)	2 (1.23)	113 (69.3)	8 (4.91)	5 (3.07)	1 (0.61)	0 (0)	0 (0)	0 (0)	0 (0)	
91 days to 14 yrs (*n* = 18)	2 (11.1)	3 (16.7)	0 (0)	8 (44.4)	2 (11.1)	2 (11.1)	1 (5.56)	0 (0)	0 (0)	0 (0)	0 (0)	
15 to 50 yrs (*n* = 324)	43 (13.3)	48 (14.8)	49 (15.1)	62 (19.1)	57 (17.6)	43 (13.3)	6 (1.85)	2 (0.62)	1 (0.31)	0 (0)	13 (4.01)	
>50 yrs (*n* = 1,001)	173 (17.3)	159 (15.9)	150 (15.0)	169 (16.9)	123 (12.3)	143 (14.3)	28 (2.80)	1 (0.10)	6 (0.60)	2 (0.20)	47 (4.70)	
Sex												<0.001*
Male (*n* = 839)	141 (16.8)	121 (14.4)	125 (14.9)	178 (21.2)	105 (12.5)	104 (12.4)	24 (2.86)	2 (0.24)	3 (0.36)	2 (0.24)	34 (4.05)	
Female (*n* = 716)	108 (15.1)	109 (15.2)	82 (11.5)	189 (26.4)	90 (12.6)	93 (13.0)	13 (1.82)	1 (0.14)	4 (0.56)	0 (0)	27 (3.77)	
Unknown^*d*^ (*n* = 1)	0 (0)	0 (0)	0 (0)	0 (0)	0 (0)	1 (100.0)	0 (0)	0 (0)	0 (0)	0 (0)	0 (0)	0.358

a NT, nontypeable.

b *, significant at a *P* value of <0.05.

c ND, not done.

d Sex not recorded.

The overall proportions of GBS isolates submitted changed significantly between historical data from 2003 to 2013 and the current data within the following age groups: EOD, LOD, adults between 15 and 50 years of age, and adults over 50 years of age (Table 2) (25). Cases of GBS infection increased in LOD (from 7.70% to 10.5%; *P = *0.006) and adults over 50 years of age (from 57.6% to 64.3%; *P* < 0.001), whereas they decreased in EOD (8.03% to 3.21%; *P* < 0.001) and adults between 15 and 50 years of age (from 25.5% to 20.8%; *P = *0.002). No GBS isolates with CPS types VII, VIII, and IX were identified in EOD, LOD, and children over the historical and current study time periods. Nontypeable CPS types decreased significantly overall (*P* < 0.001) and in both adult age groups (between 15 and 50 years of age and over 50 years of age; *P = *0.004 and *P* < 0.001, respectively) from historical to current data (25).

**TABLE 2:** Comparison of CPS types from GBS cases between historical and current data, stratified by patient age group

CPS type	Period^*a*^	% of isolates from indicated age group									
		0 to 7 days (EOD)	*P* value	8 to 90 days (LOD)	*P* value	91 days to 14 yrs	*P* value	15 to 50 yrs	*P* value	>50 yrs	*P* value
Ia	Historical	18.7	0.842	18.5	0.187	11.1	1.00	21.4	0.004*^b^	17.3	1.00
	Current	20.0		12.9		11.1		13.3		17.3	
Ib	Historical	11.9	0.701	6.15	0.546	27.8	0.423	12.5	0.361	13.2	0.09
	Current	14.0		7.98		16.7		14.8		15.9	
II	Historical	14.2	0.699	2.31	0.479	11.1	0.146	12.2	0.249	11.2	0.01*
	Current	12.0		1.23		0		15.1		15.0	
III	Historical	28.4	0.831	66.2	0.572	33.3	0.495	16.2	0.300	15.3	0.335
	Current	30.0		69.3		44.4		19.1		16.9	
IV	Historical	6.72	0.455	1.54	0.115	5.56	0.548	8.00	<0.001*	6.14	<0.001*
	Current	10.0		4.91		11.1		17.6		12.3	
V	Historical	14.2	0.452	5.38	0.322	11.1	1.00	18.4	0.06	22.0	<0.001*
	Current	10.0		3.07		11.1		13.3		14.3	
VI	Historical	1.49	0.808	0	0.372	0	0.310	1.41	0.635	1.66	0.09
	Current	2.00		0.61		5.56		1.85		2.80	
VII	Historical	0		0		0		0	0.104	0	0.327
	Current	0		0		0		0.62		0.10	
VIII	Historical	0		0		0		0.24	0.855	0.21	0.176
	Current	0		0		0		0.31		0.60	
IX	Historical	0		0		0		0.24	0.378	1.56	0.001*
	Current	0		0		0		0		0.20	
NT^*c*^	Historical	4.48	0.434	0.77	0.262	0		9.41	0.004*	11.4	<0.001*
	Current	2.00		0		0		4.01		5.00	
Overall	Historical	8.03	<0.001*	7.70	0.006*	1.08	0.829	25.5	0.002*	57.6	<0.001*
	Current	3.21		10.5		1.16		20.8		64.3	

a The historical period was from 2003 to 2013; the current period encompasses 2014 through 2020. Historical data are from reference 25.

b *, significant at a *P* value of <0.05.

c NT, nontypeable.

### Specimen sources.

Blood specimens were the most frequently submitted specimen source among the GBS isolates included in this study across all age groups (*n* = 1,147, 73.7%). Table 3 describes the proportions of GBS isolates by specimen source, stratified by age group. Cerebrospinal fluid (CSF) specimens were the most frequently submitted from cases of LOD, with 17 of 20 specimens (85.0%). Placental/cord (*n* = 14), peritoneum/dialysate (*n* = 15), and pleura (*n* = 11) specimens were the least commonly submitted sources and accounted for only 2.57% of total specimens. Deep tissue (*n* = 279) and joint/synovial fluid (*n* = 42) specimens were the second and third most frequently submitted specimen sources, respectively, and accounted for 20.6% of all specimens submitted. Adults accounted for the majority of deep tissue (15 to 50 years of age, 32.6%; over 50 years of age, 63.8%) and joint/synovial fluid (15 to 50 years of age, 31.0%; over 50 years of age, 66.7%) specimens. Twenty-eight isolates (1.80%) were from specimen sources that did not belong to any of the defined categories and were classified under Other.

**TABLE 3:** Proportions of GBS isolates by specimen source, stratified by patient age group

Specimen source (no. of isolates)	No. (%) of isolates in indicated age group					*P* value
	0 to 7 days (EOD)	8 to 90 days (LOD)	91 days to 14 yrs	15 to 50 yrs	>50 yrs	
Overall (*n* = 1,556)	50 (3.21)	163 (10.5)	18 (1.16)	324 (20.8)	1,001 (64.3)	<0.001*^a^
Blood (*n* = 1,147)	44 (3.84)	133 (11.6)	16 (1.39)	191 (16.7)	763 (66.5)	<0.001*
Cerebrospinal fluid (*n* = 20)	1 (5.00)	17 (85.0)	1 (5.00)	1 (5.00)	0 (0)	<0.001*
Placental/cord (*n* = 14)	3 (21.4)	2 (14.3)	0 (0)	9 (64.3)	0 (0)	<0.001*
Joint/synovial fluid (*n* = 42)	0 (0)	1 (2.38)	0 (0)	13 (31.0)	28 (66.7)	<0.001*
Peritoneum/dialysate (*n* = 15)	0 (0)	0 (0)	1 (6.67)	4 (26.7)	10 (66.7)	<0.001*
Pleura (*n* = 11)	0 (0)	0 (0)	0 (0)	2 (18.2)	9 (81.8)	<0.001*
Deep tissue (*n* = 279)	1 (0.36)	9 (3.23)	0 (0)	91 (32.6)	178 (63.8)	<0.001*
Other (*n* = 28)	1 (3.57)	1 (3.57)	0 (0)	13 (46.4)	13 (46.4)	<0.001*

a *, significant at a *P* value of <0.05.

### Cumulative incidence of GBS infection.

**FIG 1:**
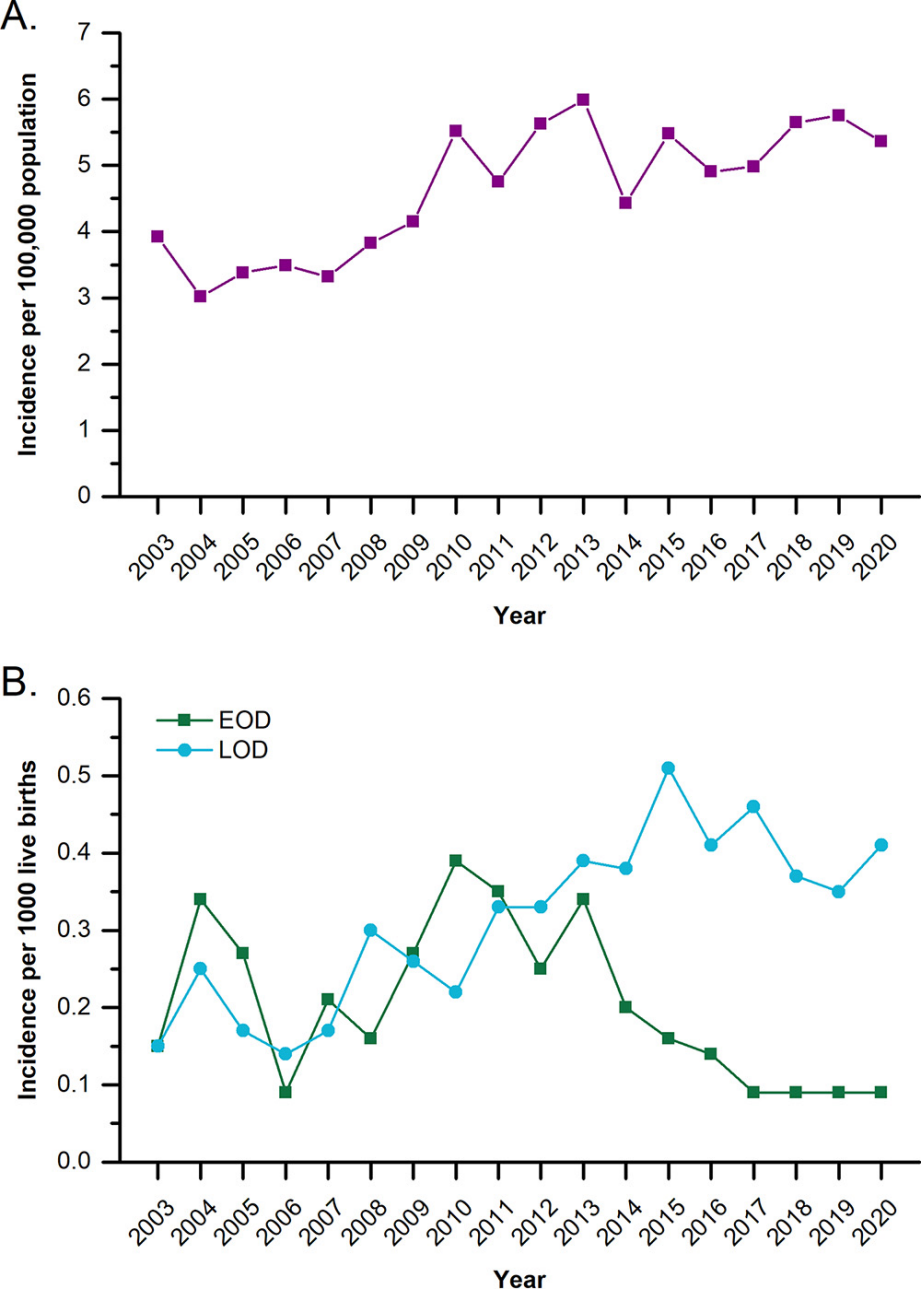
Cumulative incidence of GBS cases per 100,000 population (A) and cumulative incidences of EOD and LOD per 1,000 live births (B) over an 18-year period from 2003 to 2020 in Alberta, Canada.

The cumulative incidence of GBS infections from 2014 to 2020 increased significantly, from 4.43 per 100,000 population to 5.36 per 100,000 population (unadjusted risk ratio [RR], 1.21 [95% confidence interval {CI}, 1.06 to 1.38]), with a sharp increase in GBS infections observed in 2015 (Table 4). An overall increase in the incidence of GBS infections per 100,000 population was determined upon evaluation of historical data from 2003 up through the current study period (Fig. 1A) (25). From 2014 to 2020, 395,105 live births were recorded in the province of Alberta. The incidence of EOD associated with GBS infections decreased significantly, from 0.2 per 1,000 live births in 2014 to 0.07 per 1,000 live births in 2020 (RR, 0.35 [95% CI, 0.13 to 0.86]) (Table 4). Conversely, the incidence of LOD increased only slightly, from 0.38 per 1,000 live births to 0.41 per 1,000 live births in 2014 to 2020, respectively, and this increase was not significant (RR, 1.08 [95% CI, 0.68 to 1.72]). However, a shift in EOD and LOD incidence was observed when cumulative incidences were plotted over the 18-year period, accounting for historical and current data (Fig. 1B) (25). An overall increasing trend in both EOD and LOD incidences was observed from 2003 to 2010, but from 2011 onwards, the EOD incidence began to decrease while the LOD incidence continued to increase.

**TABLE 4:** Cumulative incidence and risk ratios of GBS cases per 100,000 population and 1000 live births

Yr	No. of cases or risk ratio (95% CI)					
	GBS cases per 100,000 population	RR	EOD cases per 1,000 live births	RR	LOD cases per 1,000 live births	RR
2014	4.43 (3.82–5.14)	Reference group	0.2 (0.1–0.37)	Reference group	0.38 (0.24–0.59)	Reference group
2015	5.48 (4.8–6.25)	1.24 (1.09–1.41)*^a^	0.16 (0.08–0.31)	0.80 (0.39–1.62)	0.51 (0.35–0.75)	1.34 (0.86–2.10)
2016	4.91 (4.27–5.64)	1.11 (0.97–1.26)	0.14 (0.07–0.29)	0.70 (0.33–1.46)	0.41 (0.26–0.62)	1.08 (0.68–1.72)
2017	4.98 (4.34–5.71)	1.12 (0.99–1.28)	0.11 (0.04–0.24)	0.55 (0.24–1.20)	0.46 (0.31–0.68)	1.21 (0.77–1.91)
2018	5.65 (4.97–6.42)	1.28 (1.12–1.45)*	0.16 (0.08–0.31)	0.80 (0.39–1.62)	0.37 (0.24–0.58)	0.97 (0.60–1.57)
2019	5.75 (5.07–6.52)	1.30 (1.15–1.47)*	0.05 (0.01–0.17)	0.25 (0.07–0.69)*	0.35 (0.22–0.56)	0.92 (0.56–1.50)
2020	5.36 (4.71–6.1)	1.21 (1.06–1.38)*	0.07 (0.02–0.19)	0.35 (0.13–0.86)*	0.41 (0.26–0.62)	1.08 (0.68–1.72)

a *, significant at a *P* value of <0.05.

### Antimicrobial resistance rates of GBS.

**FIG 2:**
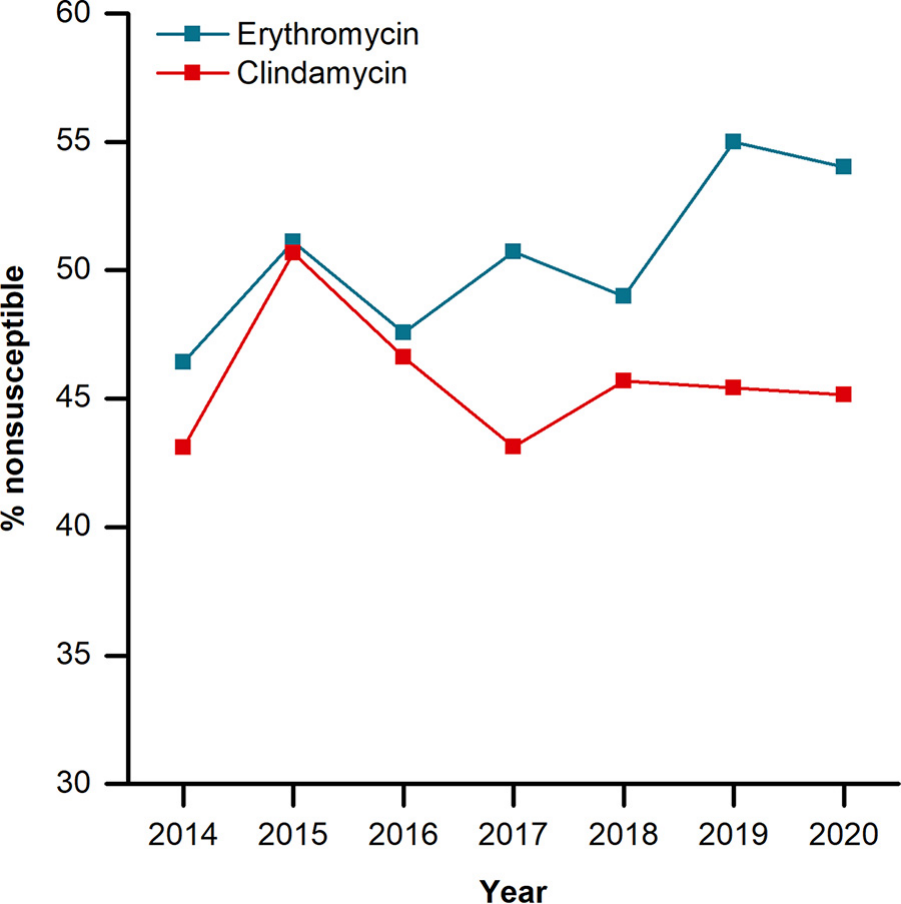
Proportions of GBS isolates exhibiting nonsusceptibility to erythromycin and clindamycin from 2014 to 2020.

Antimicrobial susceptibility testing on GBS isolates was conducted with penicillin (*n* = 1,553), vancomycin (*n* = 1,553), erythromycin (*n* = 1,543), clindamycin (*n* = 1,548), and chloramphenicol (*n* = 1,553). All GBS isolates were susceptible to penicillin and vancomycin (Table 5). Ten isolates were determined to be nonsusceptible to chloramphenicol, with 6 being intermediate and 4 being resistant. Only 1 of these 10 isolates was from a case of LOD; the remaining 9 were from adult infection cases. From 2014 to 2020, an overall increase in erythromycin nonsusceptibility in GBS isolates was observed, from 46.4% to 54.0% (Fig. 2), with the majority of nonsusceptible isolates originating from cases of adult infection (663 of 790 isolates, 83.9%). Among EOD and LOD cases, there were 29 and 88 nonsusceptible isolates, respectively. Children accounted for the remaining 10 cases with isolates nonsusceptible to erythromycin. From 2014 to 2020, the proportion of GBS isolates nonsusceptible to clindamycin rose from 43.1% to 45.1%, with a sharp increase in clindamycin nonsusceptibility (50.7%) that was observed in 2015 before it decreased to 46.6% the following year. Similar to erythromycin nonsusceptibility, adults accounted for 84.3% (*n* = 600) of GBS isolates nonsusceptible to clindamycin. There were 25 nonsusceptible isolates from cases of EOD and 78 isolates from LOD. Children represented the smallest population with isolates nonsusceptible to clindamycin, with 9 cases. Of isolates nonsusceptible to erythromycin and clindamycin, 98.48% and 98.60% were determined to be resistant to the respective antibiotic. In comparison to historical data from 2003 to 2013, the rates of nonsusceptibility to both erythromycin and clindamycin increased significantly overall during this study period (from 36.9% to 50.8% and from 21.0% to 45.8%; *P* < 0.001 and *P* < 0.001, respectively) (Table 6) (25). Specifically, erythromycin nonsusceptibility increased significantly for CPS types II (*P = *0.018), III (*P* < 0.001), and V (*P = *0.014). Except for CPS type VI, clindamycin nonsusceptibility increased significantly in all measurable CPS types (*P = *0.003 to *P* < 0.001). CPS type IV remained the most predominant CPS type associated with erythromycin and clindamycin nonsusceptibility throughout the duration of both the historical and current study periods; although erythromycin resistance did not differ significantly between study periods (*P = *0.138), clindamycin resistance increased significantly (*P = *0.038) (25).

**TABLE 5:** Proportions of GBS isolates by antimicrobial susceptibility, stratified by patient age group

Antimicrobial^*a*^	Resistance	No. (%) of isolates from indicated age group				
		0 to 7 days (EOD)	8 to 90 days (LOD)	91 days to 14 yrs	15 to 50 yrs	>50 yrs
PEN (*n* = 1,553)	Susceptible	50 (3.22)	162 (10.4)	18 (1.16)	324 (20.9)	999 (64.3)
	Intermediate	0 (0)	0 (0)	0 (0)	0 (0)	0 (0)
	Resistant	0 (0)	0 (0)	0 (0)	0 (0)	0 (0)
VAN (*n* = 1,553)	Susceptible	50 (3.2)	162 (10.4)	18 (1.16)	324 (20.9)	999 (64.3)
	Intermediate	0 (0)	0 (0)	0 (0)	0 (0)	0 (0)
	Resistant	0 (0)	0 (0)	0 (0)	0 (0)	0 (0)
ERY (*n* = 1,543)	Susceptible	21 (1.36)	73 (4.73)	10 (0.52)	143 (9.27)	508 (32.9)
	Intermediate	1 (0.06)	0 (0)	0 (0)	0 (0)	11 (0.71)
	Resistant	28 (1.81)	88 (5.70)	10 (0.65)	180 (11.7)	472 (30.6)
CLI (*n* = 1,548)	Susceptible	25 (1.61)	84 (5.43)	9 (0.58)	161 (10.4)	557 (36.0)
	Intermediate	0 (0)	3 (0.19)	0 (0)	0 (0)	7 (0.45)
	Resistant	25 (1.61)	75 (4.84)	9 (0.58)	162 (10.5)	431 (27.8)
CHL (*n* = 1,553)	Susceptible	50 (3.22)	161 (10.4)	18 (1.16)	322 (20.7)	992 (63.9)
	Intermediate	0 (0)	1 (0.06)	0 (0)	0 (0)	5 (0.32)
	Resistant	0 (0)	0 (0)	0 (0)	2 (0.13)	2 (0.13)

a PEN, penicillin; VAN, vancomycin; ERY, erythromycin; CLI, clindamycin; CHL, chloramphenicol.

**TABLE 6:** Comparison of CPS types from GBS cases between historical and current data, stratified by erythromycin and clindamycin nonsusceptibility

CPS type	Period^*a*^	% of isolates that were nonsusceptible to:			
		Erythromycin	*P* value	Clindamycin	*P* value
Ia	Historical	29.5	0.060	4.49	<0.001*^b^
	Current	37.0		13.7	
Ib	Historical	33.3	0.144	25.4	0.002*
	Current	40.0		39.6	
II	Historical	41.7	0.018*	31.0	<0.001*
	Current	53.6		50.2	
III	Historical	26.7	<0.001*	17.0	<0.001*
	Current	51.0		49.0	
IV	Historical	80.2	0.138	77.4	0.038*
	Current	86.7		86.7	
V	Historical	41.3	0.014*	25.8	<0.001*
	Current	52.3		49.2	
VI	Historical	37.5	0.269	29.2	0.501
	Current	24.3		21.6	
VII	Historical	0	0	0	0
	Current	33.3		33.3	
VIII	Historical	0	0	0	0
	Current	14.3		14.3	
IX	Historical	25.0	0	31.3	0
	Current	0		0	
NT^*c*^	Historical	33.3	0.419	22.0	0.003*
	Current	39.1		42.2	
Overall	Historical	36.9	<0.001*	21.0	<0.001*
	Current	50.8		45.8	

a The historical period was from 2003 to 2013; the current period encompasses 2014 through 2020. Historical data are from reference 25.

b *, significant at a *P* value of <0.05.

c NT, nontypeable.

## DISCUSSION

Globally, GBS is a leading cause of neonatal morbidity and mortality (8, 26, 29–31). Approximately 30% of women are naturally colonized with GBS, including those that are pregnant (32). A previous study found that GBS colonization in pregnant women can be transient, occurring in approximately 28% to 50% of this population (33). With GBS colonization comes the opportunity for vertical transmission of GBS from mother to newborn, which can occur through ascending infection *in utero* or during delivery (9, 10). With the implementation of culture-based screening and administration of intrapartum chemoprophylaxis in the province of Alberta, the incidence of EOD per 1,000 live births has decreased significantly (from 0.2 in 2014 to 0.07 in 2020), as described in this study. Interestingly, in our previous study, Alhhazmi et al. observed an increase in EOD incidence over the 11-year period from 2003 to 2013 for unknown reasons, despite chemoprophylaxis measures having been in place (25). Although the incidence of EOD has now decreased over time, the incidence of LOD per 1,000 live births has increased (from 0.38 in 2014 to 0.41 in 2020) in the province. This finding is in agreement with increasing LOD rates also seen in the United States, as intrapartum chemoprophylaxis mainly serves to mitigate the burden of EOD only (32, 34). In the case of LOD, newborns may acquire GBS from sources not limited to colonization during delivery, including prolonged stays in neonatal intensive care units, invasive medical procedures, poor infection control practices, and breast milk (35–37).

CPS type III continues to be a dominating CPS type in Alberta, corresponding with our previous report on GBS epidemiology in Alberta from 2003 to 2013 and in other parts of the world (25, 38–40). Furthermore, CPS type III also accounts for the majority of GBS infections in EOD, LOD, and children, while in adults, the proportions of Ia, Ib, II, III, and V are comparable. This finding is expected, as CPS type III is most widely associated with cases of neonatal GBS disease (24). Previously, we reported that CPS types V and Ia were the most common CPS types in Alberta after type III. However, from the surveillance period of 2014 to 2020 captured in this study, CPS type Ib is now the third most frequently identified type of GBS after Ia, with V as the fourth most common. In 2019, GBS type Ib just overtook type III as the most common CPS type, with 20.3% of GBS isolates being type Ib and 19.5% being type III for all ages. Large proportions of CPS type Ib among isolate collections have been reported in East Asian and South American populations (24, 41, 42). Interestingly, recent data from the Active Bacterial Core surveillance program in the United States found CPS type Ia to be the most frequent CPS type, with CPS type Ib the fifth most frequent (43). Similarly, in a study by Teatero et al. from Toronto, Canada, type Ib was the fifth most common CPS type, behind types III, V, Ia, and II (22). Continual surveillance of GBS epidemiology and CPS typing are necessary to determine if the distribution of CPS types in the province will continue to shift in this direction.

With the departure from double immunodiffusion serotyping and adoption of a real-time PCR assay for CPS typing, the number of nontypeable GBS isolates has drastically decreased, indicating an improvement in specificity from this technique. Prior to 2017, an average of 19 isolates per calendar year were nontypeable. Using the real-time PCR assay, only 1 nontypeable GBS isolate was reported in our study for each year in 2017, 2018, and 2019, and none in 2020. Thus, a more accurate depiction of CPS distribution in the province is now available for subsequent surveillance studies. It is possible that changes in the capsule gene operon, such as gene loss or mutation, may account for the remaining nontypeable CPS isolates seen in our study (44, 45).

In our previous study, we documented the introduction and expansion of CPS type IV in Alberta and observed a 10-fold increase (1.62% to 10.3%) in type IV isolates during the 11-year surveillance period (25). Similar results were reported in other provinces across Canada (46–48). In the current study, CPS type IV has increased to now account for 13.9% of GBS isolates in 2020. Significant increases in type IV GBS isolates were observed in cases of adult infections in Alberta compared to historical data. Twofold increases in type IV GBS were seen in adults 15 to 50 years of age (from 8.00% to 17.6%) and in those over 50 years of age (6.14% to 12.3%), suggesting an association with invasive GBS disease in nonpregnant patients. Type IV GBS is of additional concern, as capsular switching from type III to IV has been reported (49, 50).

An overall increase in GBS disease in the Alberta population is suggested from the updated data set. Over our 7-year study period, the cumulative incidence of GBS per 100,000 population increased by 21% (from 4.43 to 5.36; RR, 1.21 [95% CI, 1.06 to 1.38]) and is on trend with the 50% increase that was observed in our previous study over an 11-year period. A significant increase in GBS cases was observed in 2015, when an incidence of 5.48 per 100,000 population was calculated. Interestingly, there was no known GBS outbreak that occurred during this time, and the distribution of GBS cases between the age groups did not differ from other years. Thus, it is unknown whether the unexpected increase in incidence in 2015 is attributable to a rise in GBS disease in the Alberta population or a coincidental increase in the submission of isolates to the diagnostic laboratory. Going forward, it will be of value to determine if GBS disease incidence continues on this upward trajectory.

The increase in erythromycin and clindamycin nonsusceptibility over the 7-year period in this study is worthy of concern. Compared to historical data, the overall rate of nonsusceptibility to erythromycin increased by 1.38-fold (from 36.9% to 50.8%) and to clindamycin by 2.18-fold (from 21.0% to 45.8%). Only 1.52% and 1.40% of the nonsusceptible isolates displayed intermediate susceptibility to erythromycin and clindamycin, respectively, indicating that the vast majority of nonsusceptible GBS isolates are resistant to these antibiotics. The majority of nonsusceptible GBS isolates were associated with adult infections (erythromycin, 83.9%, and clindamycin, 84.3%). Taking into consideration that CPS types II, III, and V are 3 commonly identified GBS types in Alberta that had significant increases in erythromycin nonsusceptibility, all surpassing 50%, the option of using erythromycin as a penicillin alternative in intrapartum chemoprophylaxis may require reevaluation. In regard to clindamycin nonsusceptibility, significant increases were observed for all measurable CPS types, suggesting that clindamycin nonsusceptibility is more ubiquitous among the different CPS types. However, almost all GBS isolates that were nonsusceptible to clindamycin were also nonsusceptible to erythromycin (*n* = 689, 96.77%). Similar rates of resistance to erythromycin and clindamycin have been observed in various parts of the world, including Canada, the United States, Europe, China, and Taiwan (28, 43, 47, 51–55). Resistance to erythromycin and clindamycin is primarily associated with *ermB*, *ermTR*, or *mef* (56). Fortunately, a number of GBS vaccine candidates are currently in clinical trials. A promising candidate is a hexavalent CPS conjugate vaccine currently in clinical trials (ClinicalTrials.gov accession number NCT03170609), which targets CPS types Ia, Ib, II, III, IV, and V. These 6 types have been shown to be the most prevalent CPS types in circulation globally (40), and the hexavalent candidate has completed phase I/II trials and trials in pregnant women (57). As antimicrobial resistance to penicillin alternatives continues to rise, a GBS vaccine for pregnant women would significantly aid in reducing the burden of neonatal GBS disease and mortality.

In conclusion, we present an update on invasive GBS disease epidemiology in Alberta, Canada, from 2014 to 2020. During this 7-year surveillance period, we observed a change in the predominant CPS types circulating in the province. Although CPS type III remains the most frequently identified type, the overall proportions of CPS types Ib, II, and IV have also risen. We saw significant increases in GBS disease and LOD incidence but a significant decrease in EOD incidence, suggesting that the use of intrapartum chemoprophylaxis treatment is preventing disease. However, the rates of nonsusceptibility of GBS to erythromycin and clindamycin have continued to increase over the 18-year period that GBS disease has been monitored across Alberta. On-going surveillance and epidemiological studies are crucial to improve maternal and neonatal invasive GBS disease treatment and can help lead to the prevention of invasive GBS disease.

## MATERIALS AND METHODS

### Case definitions and bacterial isolates.

In 2011, neonatal streptococcal disease became notifiable to public health authorities within the province of Alberta upon clinical presentation and laboratory confirmation of GBS via isolation of the organism or detection of nucleic acid from a sterile site (blood, cerebrospinal fluid [CSF], deep tissue, bone, pleural, peritoneal, pericardial, or joint fluid specimens). As a result, all invasive GBS isolates were forwarded to the Alberta Public Health Laboratory for CPS typing. Neonatal GBS disease was classified as early-onset disease (EOD), occurring between 0 and 7 days, or late-onset disease (LOD), which occurs between 8 and 90 days of age. The demographics of patient age, sex, specimen collection date, and anatomical source were retrieved from the Alberta Precision Laboratories—Public Health Laboratory Information System for epidemiological analyses.

This study spanned from 1 January 2014 to 31 December 2020, from which time period a total of 1,556 GBS isolates from cases of infection in neonates (EOD and LOD), children (91 days to 14 years), and adults (15 to 50 years and >50 years) were included. All duplicate isolates originating from the same patient within the same calendar year, regardless of anatomical collection source, were excluded from the analyses. Of the 213 isolates from neonatal infections, 50 were categorized as EOD, and the remaining 163 isolates were classified as LOD. Infections in children and adults accounted for the remaining 18 and 1,523 isolates, respectively. The population in Alberta increased from 4,083,652 to 4,421,887 from 2014 to 2020, and the number of live births increased from 55,458 to 56,618.

### Capsular polysaccharide typing.

Until January 2017, CPS typing for CPS types Ia, Ib, and II through VIII was performed at the Alberta Public Health Laboratory using a double immunodiffusion method with CPS-specific antisera developed in rabbits (25, 58, 59). From January 2017 onwards, a real-time PCR assay utilizing hydrolysis probes developed by Alhhazmi et al. (CPS Ia, Ib and II-VIII) and Imperi et al. (CPS IX) that target the CPS-determining region was adopted for all CPS typing conducted at the Alberta Public Health Laboratory (60, 61). In brief, the typing algorithm consisted of 5 multiplex PCRs for the detection of *cps* genes *cpsH*, *cpsJ*, *cpsK*, *cpsG*, *cpsN*, *cpsO*-V, *cpsI*, *cpsM*, *cpsR*, and *cpsO*-IX, which correspond to CPS types Ia, Ib, and II through IX, respectively.

### Antimicrobial susceptibility testing.

Antimicrobial susceptibility testing for penicillin, vancomycin, erythromycin, clindamycin, and chloramphenicol resistance was conducted on all isolates by disk diffusion (Oxoid Limited, Nepean, ON, Canada) at the Alberta Public Health Laboratory. Interpretations of zone diameter breakpoints were made according to the Clinical and Laboratory Standards Institute (CLSI) M100 Performance Standards for Antimicrobial Susceptibility Testing (62). The numbers of nonsusceptible isolates interpreted as intermediate and resistant were analyzed for each antimicrobial agent.

### Data analysis.

Descriptive statistics for CPS serotypes of GBS and specimen sources were conducted using proportional analyses for a binary outcome. GBS CPS serotypes were stratified by patient demographics (year of testing, patient age group, and sex), and specimen sources were stratified by patient age group. The chi-square test was used to analyze statistical differences within stratified groups. CPS type distributions across age groups and erythromycin/clindamycin susceptibilities were compared between historical (2003 to 2013) and current (2014 to 2020) time periods using the two-proportion *z* test. The cumulative incidence of GBS was analyzed per 100,000 population, while EOD and LOD incidences were analyzed per 1,000 live births from 2014 to 2020. To compare cumulative incidences between years, unadjusted risk ratios (RR) were analyzed, encompassing test year and incidence proportions, with 2014 as the reference group. The cutoff for statistical significance was α = 0.05, and all analyses were performed using STATA statistical software version 16.0 (63). All graphs were produced with Origin Pro 2016 (OriginLab, Northampton, MA, USA).
